# Supplementary figures and images for: Effect of closed and permanent stoma on disease course, psychological well-being and working capacity in Swiss IBD cohort study patients
Source: PLoS One. 2022 Sep 16;17(9):e0274665. doi: 10.1371/journal.pone.0274665 (PMC9481029; doi:10.1371/journal.pone.0274665)

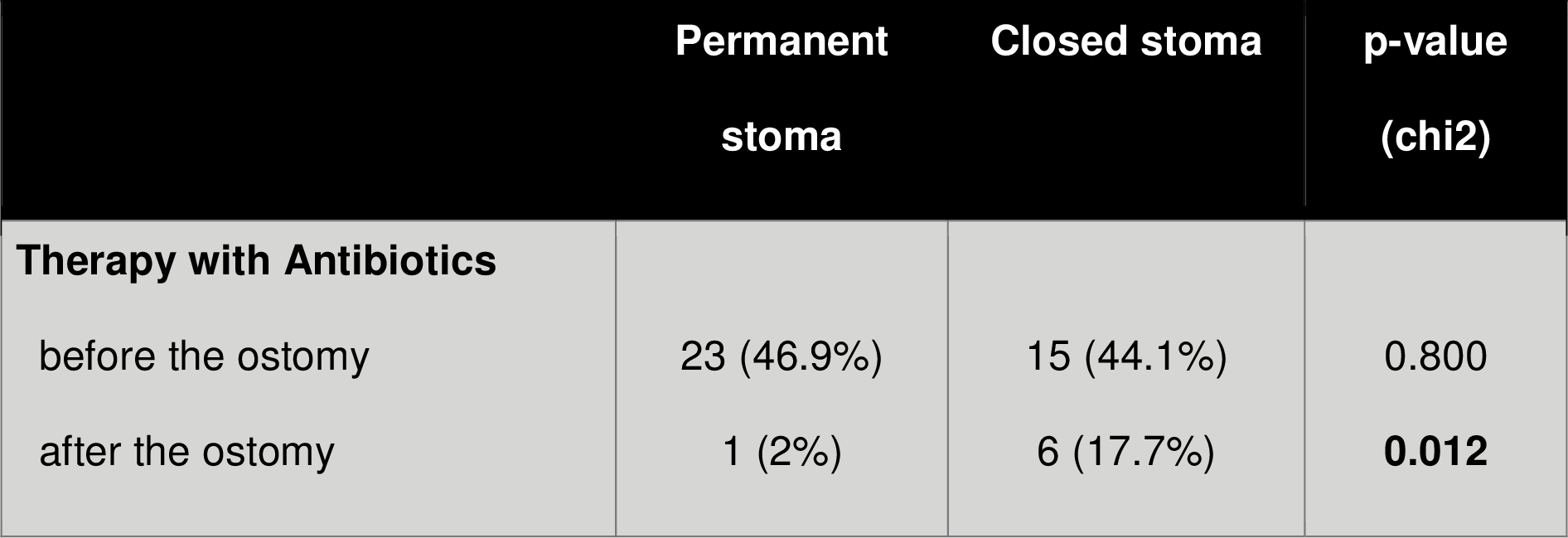

Supplement: S1 Fig — (TIF) [file pone.0274665.s001.tif]

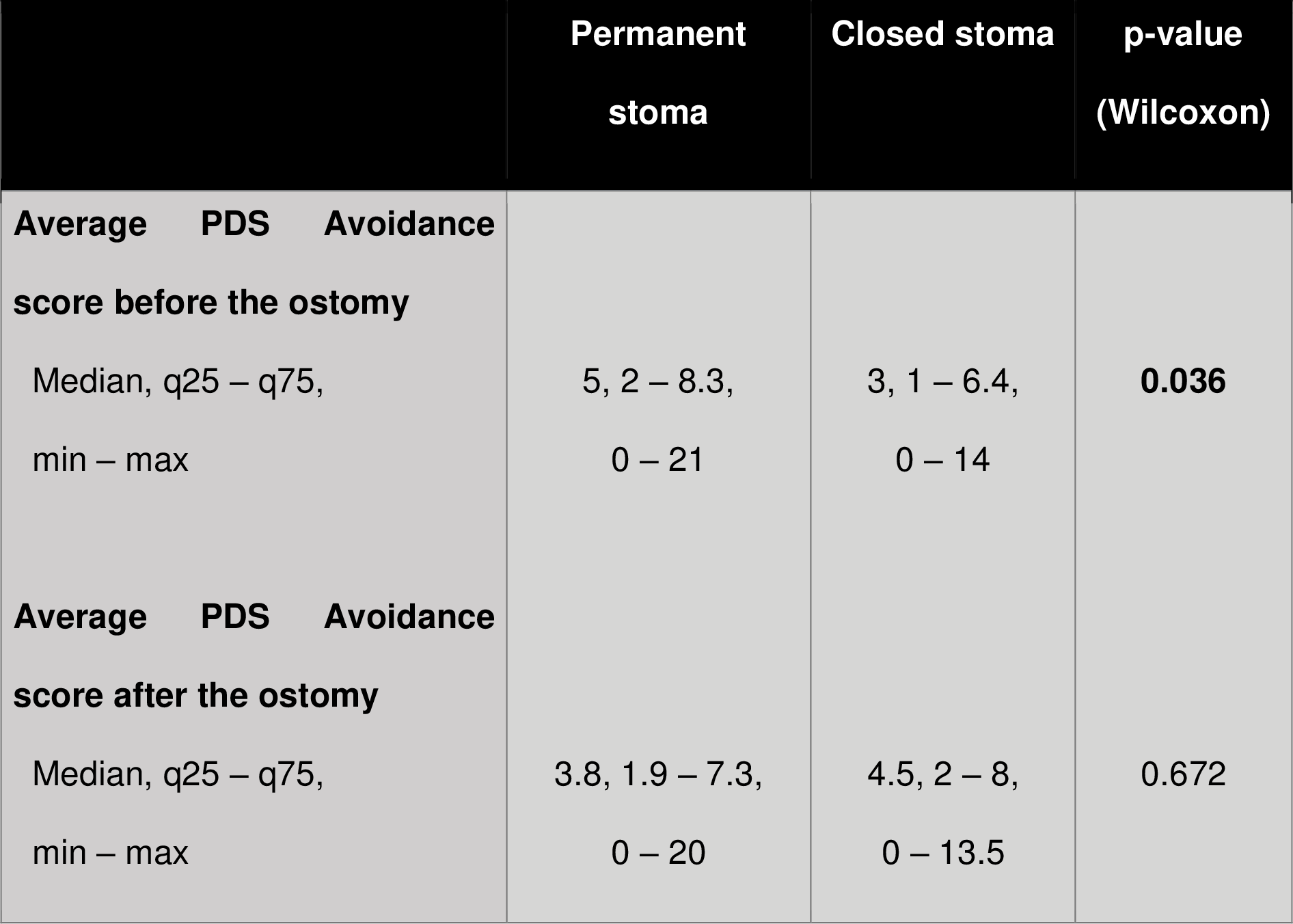

Supplement: S2 Fig — (TIF) [file pone.0274665.s002.tif]

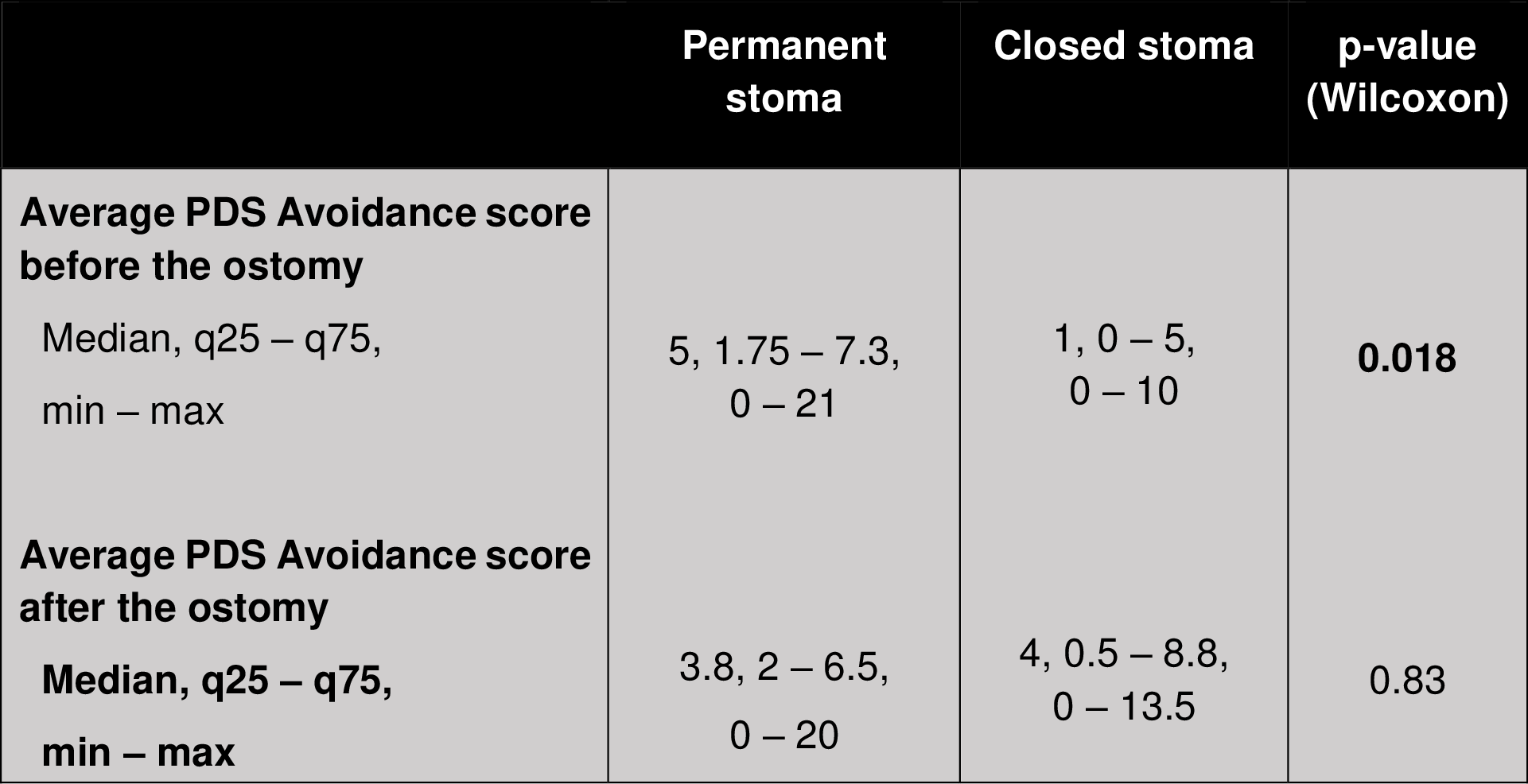

Supplement: S3 Fig — (TIF) [file pone.0274665.s003.tif]

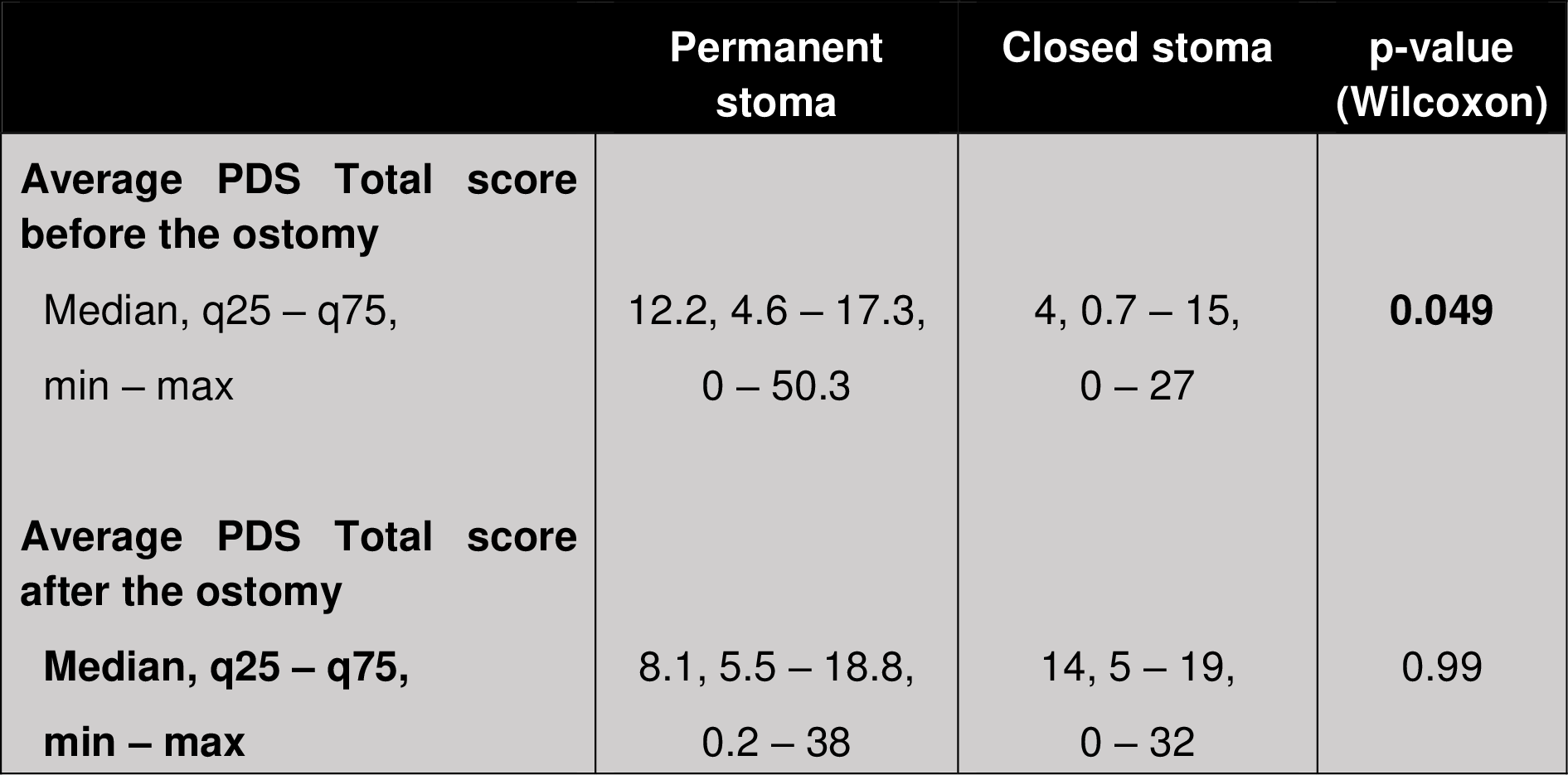

Supplement: S4 Fig — (TIF) [file pone.0274665.s004.tif]

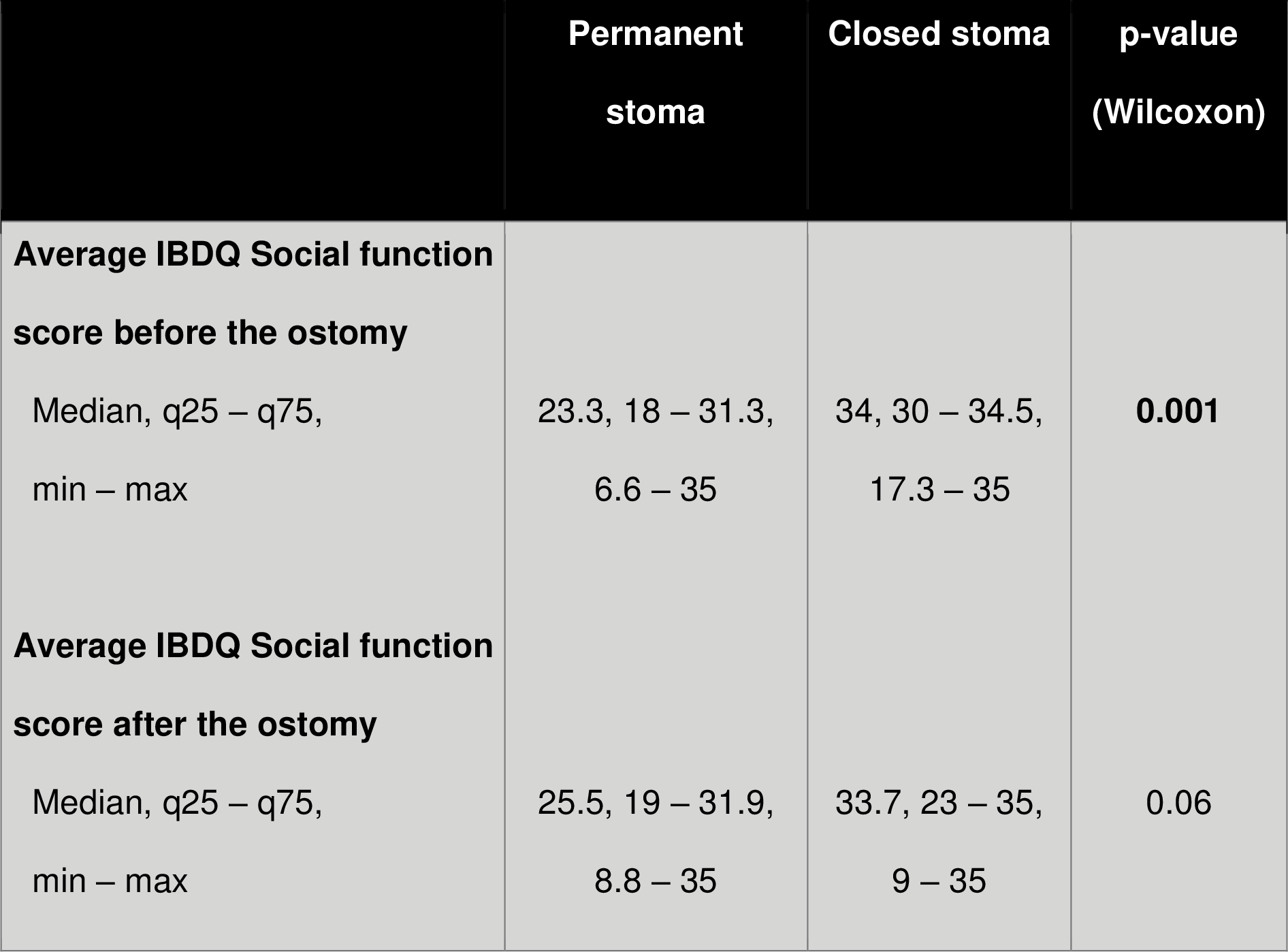

Supplement: S5 Fig — (TIF) [file pone.0274665.s005.tif]

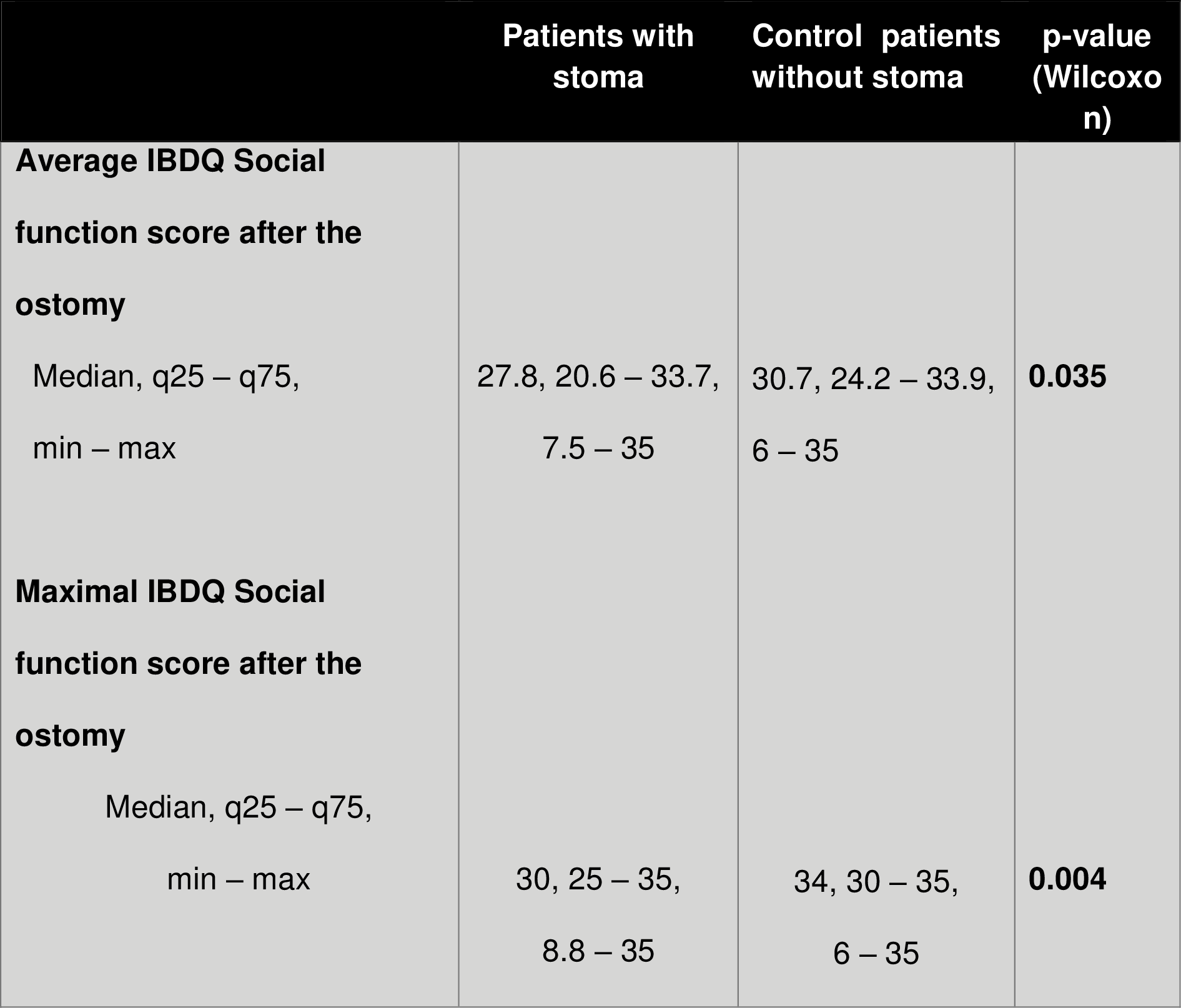

Supplement: S6 Fig — (TIF) [file pone.0274665.s006.tif]

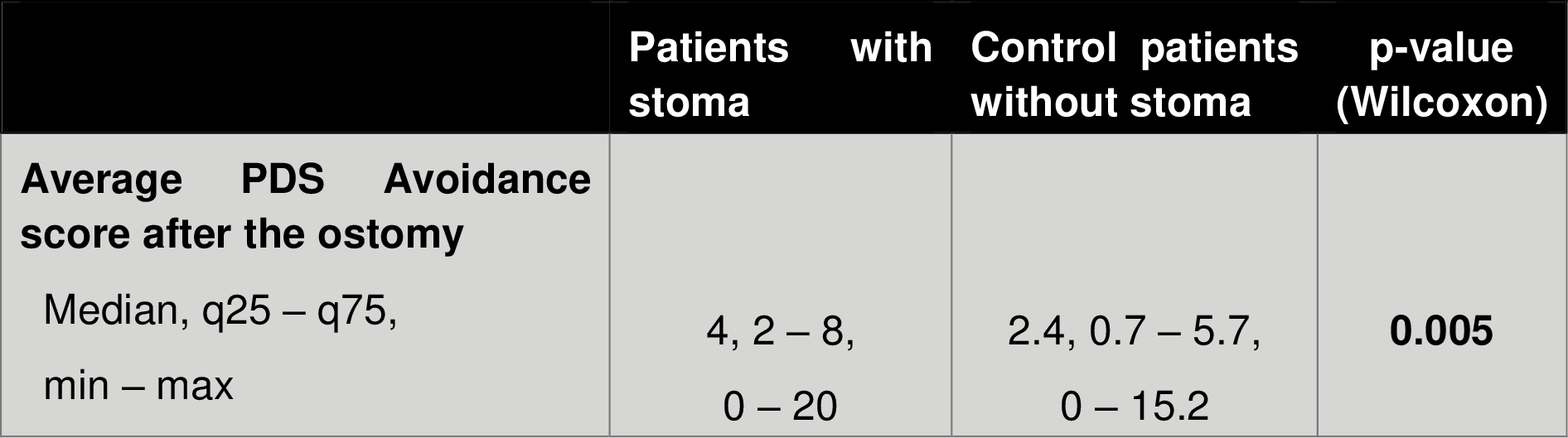

Supplement: S7 Fig — (TIF) [file pone.0274665.s007.tif]

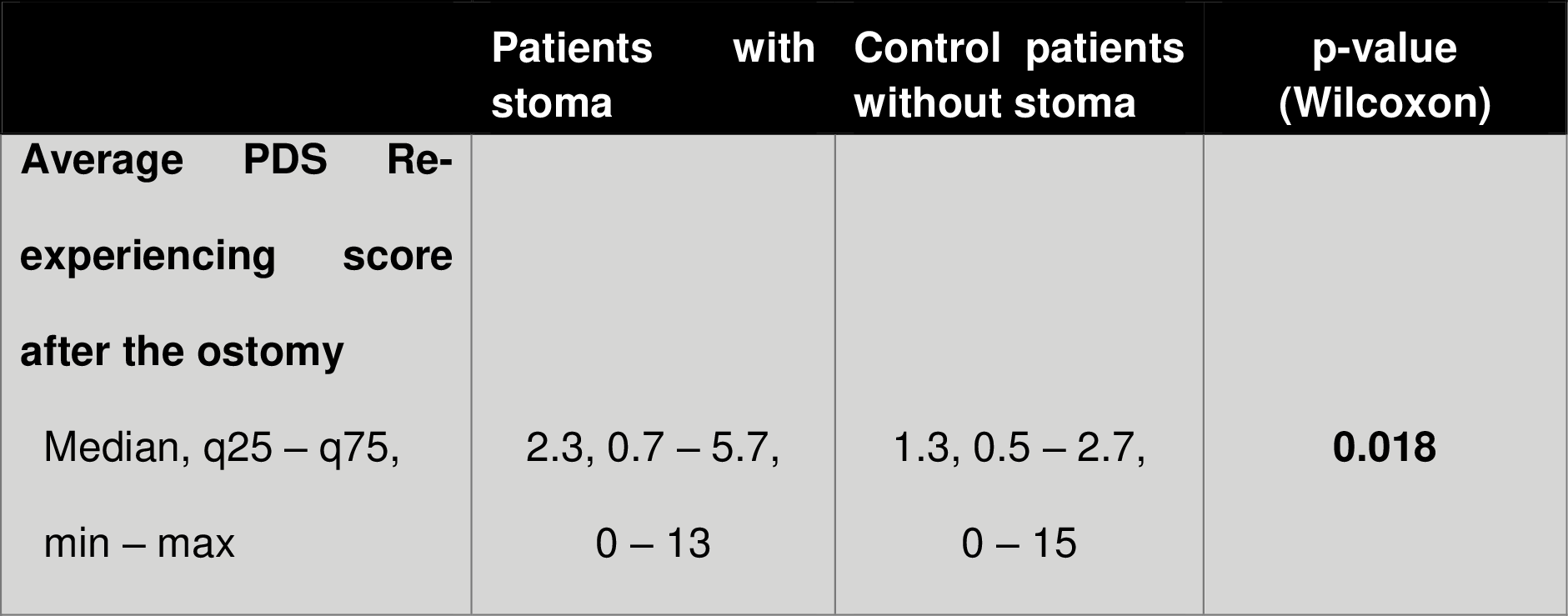

Supplement: S8 Fig — (TIF) [file pone.0274665.s008.tif]
